# P379: Contribution of environmental measures in the implementation of a programme of infection control in Africa: an example of Senegal

**DOI:** 10.1186/2047-2994-2-S1-P379

**Published:** 2013-06-20

**Authors:** N Mamadou, B Ndoye, M SECK

**Affiliations:** 1 PRONALIN, Ministry of Health, Dakar, Senegal; 2 ICAN/RIPAQS, Dakar, Senegal

## Introduction

In the context of strengthening the health system, the PRONALIN implemented in Senegal since 2004, has received funding from the Global Fund to operationalize its activities in all health facilities in the country. The objective is to assess the role of environmental measures in order to determine their contribution to the implementation of a program of infection control.

## Objectives

To evaluate the performance of the environmental aspects have an impact on patient safety.

## Methods

A concrete roadmap (with organizational measures and technical measures targeting certain basic processes priority) has been proposed care facilities. Grid supervision to enable an assessment activities was then used during formative supervisions biannual regularly conducted in all health facilities from January 2010 to December 2012.

## Results

The organizational measures were performed in 96.13% of cases. Among the measures proposed techniques, waste management care comes first with a rate of 96% where the activity is partially completed, followed by hand hygiene (61%), then with 55% biocleaning. Other technical activities follow far behind.

## Conclusion

In addition to hand hygiene has received wide campaign for many years, are the environmental measures that have given the best performance of technical measures.

In a context of shortage of human resources and material environmental measures remain the most technical measures to reach health facilities. This is of great interest because they allow a clear and noticeable improvement of working conditions, introduces personal teamwork, and all these positive results are a motivation for further work.

## Disclosure of interest

None declared

